# Spatiotemporal sensitivity of mesoderm specification to FGFR signalling in the *Drosophila* embryo

**DOI:** 10.1038/s41598-021-93512-1

**Published:** 2021-07-08

**Authors:** V. Yadav, N. Tolwinski, T. E. Saunders

**Affiliations:** 1 Mechanobiology Institute, National University of Singapore, Singapore, Singapore; 2 Yale-NUS, National University of Singapore, Singapore, Singapore; 3 Department of Biological Sciences, National University of Singapore, Singapore, Singapore; 4 Institute of Molecular and Cell Biology, A*Star, Singapore, Singapore; 5 Warwick Medical School, University of Warwick, Coventry, UK

**Keywords:** Biological techniques, Developmental biology

## Abstract

Development of the *Drosophila* embryonic mesoderm is controlled through both internal and external inputs to the mesoderm. One such factor is Heartless (Htl), a Fibroblast Growth Factor Receptor (FGFR) expressed in the mesoderm. Although Htl has been extensively studied, the dynamics of its action are poorly understood after the initial phases of mesoderm formation and spreading. To begin to address this challenge, we have developed an optogenetic version of the FGFR Heartless in *Drosophila* (Opto-htl). Opto-htl enables us to activate the FGFR pathway in selective spatial (~ 35 μm section from one of the lateral sides of the embryo) and temporal domains (ranging from 40 min to 14 h) during embryogenesis. Importantly, the effects can be tuned by the intensity of light-activation, making this approach significantly more flexible than other genetic approaches. We performed controlled perturbations to the FGFR pathway to define the contribution of Htl signalling to the formation of the developing embryonic heart and somatic muscles. We find a direct correlation between Htl signalling dosage and number of Tinman-positive heart cells specified. Opto-htl activation favours the specification of Tinman positive cardioblasts and eliminates Eve-positive DA1 muscles. This effect is seen to increase progressively with increasing light intensity. Therefore, fine tuning of phenotypic responses to varied Htl signalling dosage can be achieved more conveniently than with other genetic approaches. Overall, Opto-htl is a powerful new tool for dissecting the role of FGFR signalling during development.

## Introduction

Morphogenetic events are tightly controlled in space and time^1,2^. Throughout development, cells propagate, migrate, and differentiate to form specialised structures in a highly regulated manner. Such organisation within the embryo is dictated by numerous signalling events, some of which are highly conserved across organisms^3–5^. Many of these signalling pathways interact with each other, generating complex network interactions and are interpreted differently by cells based on their competence to respond^5–8^. These signalling networks are regulated in both space and time throughout morphogenesis to ensure that development is robust and reproducible^9–11^.

A crucial signalling pathway in regulating cell behaviour during development is the highly conserved Fibroblast Growth Factor Receptor (FGFR) pathway^12^. FGFRs are transmembrane proteins that belong to the receptor tyrosine kinase family. Ligand-binding leads to FGFR homo-dimerisation, initiating trans-phosphorylation events which ultimately activate transcription of several target genes^13^. This transcriptional response regulates different cellular responses, such as changes in cell morphology, proliferation, adhesion, migration and differentiation^14–18^. Due to their involvement in key cellular processes, FGFRs when mutated can promote cancer development and progression^19,20^.

There are two known FGFRs in *Drosophila* that control distinct developmental processes, *heartless* (*htl*) and *breathless (btl)*. These bind three ligands in total—Pyramus, Thisbe and Branchless^21,22^. This compares with four known FGFRs and at least 22 associated ligands in humans that bind each other in several different combinations^23^. The small number of receptor-ligand combinations makes *Drosophila* an excellent system to explore the basic interactions underlying FGFR action. In *Drosophila, htl* expression is important for proper development of several mesoderm derived tissues, including the heart and muscles^24–26^. *btl* expression is required for proper morphogenesis of the trachea^27,28^. Both Htl and Btl are essential in driving proper migration of mesodermal, glial, and tracheal cells^29^. For instance, Htl plays a role in the spreading of the mesoderm over the ectoderm to form a monolayer during early stages of embryogenesis^30–32^. Uniform spreading of the mesoderm is crucial for proper cell-fate specification of different cell types within the mesoderm at later stages^21,22^. In *htl* mutants, mesoderm cells fail to undergo proper spreading and form irregular and multilayer arrangements. This lack of structure prevents mesodermal cells from receiving precise spatial cues from the ectoderm. Later in development, Htl is also involved in the specification of different cell types derived from the mesoderm^33^. *htl* null mutants lack precursors of pericardial and heart cells, have defects in visceral mesoderm, and show reduced, irregular muscle patterns^21,22,24^.

The role of FGFR in cell fate specification has been extensively studied^34–36^. While previous work has provided detailed insights into how Htl controls the movement of mesodermal cells during the spreading phase^37^, the in vivo dynamics of Htl action within the developing mesoderm remain elusive after the initial stages of spreading. Genetic perturbations of *htl* offer only a limited exploration of the spatiotemporal range of Htl activity. In recent years, the use of optogenetics to tune signalling pathway responses has become a powerful tool in vivo^10,38–40^. Optogenetic approaches enable precise spatiotemporal tuning of target activity, enabling in vivo signalling dynamics to be dissected.

Here, we utilised an optogenetic tool (termed Opto-htl) to activate Htl signalling in a spatiotemporally controlled manner during *Drosophila* embryo development. Upon illumination with 488 nm light, Opto-htl functions as a constitutively active receptor, capable of activating downstream factors of the FGFR pathway, such as the extracellular signal regulated kinase (Erk). Opto-htl restored a significant number of heart cells within a *htl* mutant upon light activation, though it did not fully rescue the mutant phenotype. Constitutive activation of Opto-htl in the mesoderm of wild-type embryos led to several developmental defects, the severity of which varied with changes in light intensity, timing, and spatial organisation of the light exposure. We identified a time window of sensitivity to FGFR over-activation (stage 10 till late stage 12 of embryogenesis), illumination during which was both necessary and sufficient to induce the phenotypic defects. Together, these results demonstrate sensitivity of the Htl-dependent processes (particularly heart formation) to over-activation of Htl.

## Results

### Opto-htl can stimulate FGFR activity

**Figure 1:**
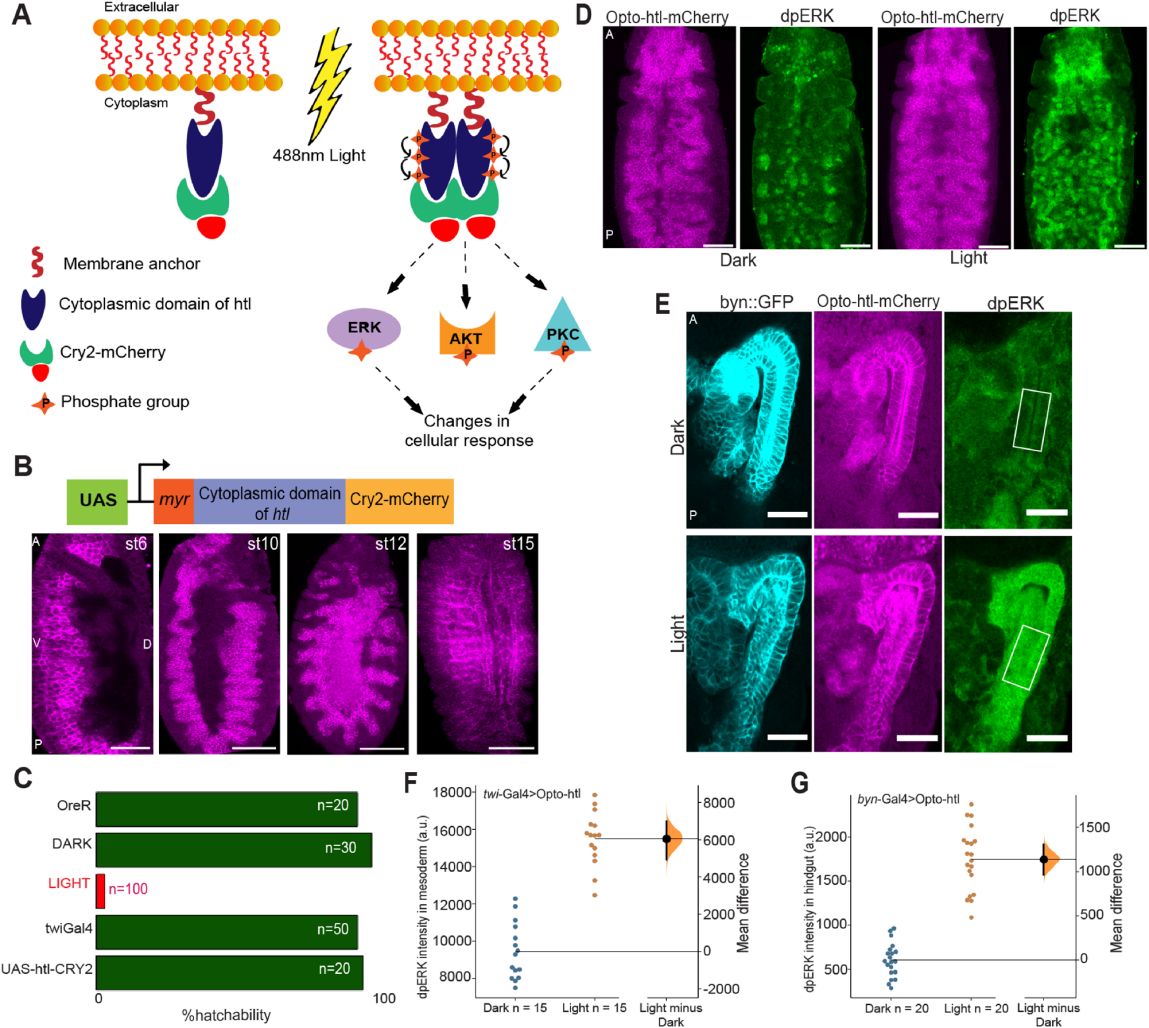
Opto-htl expression and activation in different tissues. (**A**) Schematic showing design and activation of Opto-htl. Exposure to 488 nm light induces CRY2 clustering leading to activation of the (intracellular) membrane-bound receptor and eventually generating a cellular response via phosphorylation of various downstream target molecules. (**B**) *twi*::Gal4 > Opto-htl embryos stained with anti-mCherry antibody showing the expression of the construct at various stages. (**C**) Hatching rate assay for different genetic conditions. OreR = OregonR embryos under illumination, DARK = *twi*::Gal4 > Opto-htl embryos kept under illumination with amber paper to block 488 nm wavelengths throughout development, LIGHT = *twi*::Gal4 > Opto-htl embryos kept under illumination throughout development. *twi*::Gal4 embryos represent the Gal4 driver alone and UAS-htl-Cry2-mCherry represents Opto-htl embryos with no Gal4 driver kept under similar illumination conditions. (**D**) *twi*::Gal4 > Opto-htl embryos fixed and stained at late stage 10/early stage 11 for dpErk under dark and light conditions. (**E**) *Byn*::Gal4 > Opto-htl embryos fixed and stained at stage 16 for dpErk under dark and light conditions (scale bar 25 mm). (**F**) dpErk intensity differences in the mesoderm at late stage 10 between *twi*::Gal4 > Opto-htl embryos kept under dark versus light. (**G**) dpErk intensity differences in the hindgut between *Byn*::Gal4 > Opto-htl embryos under dark and light conditions in stage 16 measured in the region shown in (**E**). Scale bar = 50 μm unless stated otherwise. A = Anterior, P = Posterior, D = Dorsal, V = Ventral view. In (**F**,**G**), the black bar represents the 95% confidence interval with the Bootstrap distribution shown in orange and n represents number of embryos for each condition^68^.

To generate an in vivo optogenetic tool for FGFR activation, we utilised Cryptochrome2 (CRY2), a light-interacting molecule that undergoes oligomerisation upon exposure to 488 nm blue light^41^. The cytoplasmic domain of *htl* was fused with CRY2-mCherry and the resulting fusion protein (termed Opto-htl) was anchored to the membrane by a myristoylation (myr) signal sequence^42^. Light exposure induces oligomerisation of CRY2, bringing receptor molecules together and triggering a phosphorylation cascade, which should lead to ligand-independent activation of target genes downstream of the receptor (Fig. 1A).

We used *twi*::Gal4 to drive Opto-htl (*twi*::Gal4 > UAS-htl-CRY2-mCherry) in the mesoderm. We validated that our construct was expressed in the mesoderm and mesoderm-derived tissues at different stages throughout embryogenesis (Fig. 1B). Live imaging showed that expression levels remained low until stage 10 but subsequently increased and were maintained in the mesoderm throughout embryogenesis (Movie S1). *twi*::Gal4 > Opto-htl embryos maintained in the dark throughout embryogenesis hatched with similar frequency to wild-type (OreR) embryos (Movie S2). However, upon constant light exposure (Methods) from stage 5 onwards, there was a significant decrease in hatching rate and 97% of embryos died before hatching (Fig. 1C). These embryos developed up until the later stages of embryogenesis before dying unhatched (Movie S3). In all other conditions, including wild-type embryos kept under the same light exposure, over 90% of embryos hatched (Fig. 1C).

Next, we tested the constitutive activation of the FGFR pathway via Opto-htl. We used the phosphorylation of Erk as a readout for the MAPK/Erk pathway downstream of FGFR^43^. Erk activation to doubly phosphorylated Erk (dpErk) leads to transcription of several target genes that regulate growth and proliferation^44^. We tested for differences in the levels of dpErk in Opto-htl embryos kept under dark and light conditions. We illuminated *twi*::Gal4 > Opto-htl embryos up until the end of germ band elongation (late stage 10 / early stage 11), then fixed and stained them with anti-dpErk antibody alongside embryos kept in the dark. We observed an increase in dpErk levels in embryos exposed to light as compared to the ones fixed and stained at a similar stage in the dark (Fig. 1D). We also tested Erk activation in the hindgut epithelium (ectoderm-derived) which does not express high levels of dpErk in wild-type conditions. We used *byn:*:Gal4 to express Opto-htl in the hindgut epithelium and again tested for dpErk levels under dark and light conditions (Fig. 1E). Comparing the dpErk intensity for dark and light conditions in both tissues, dpErk levels were significantly increased upon light activation (Fig. 1F–G). We conclude that Opto-htl can be used to induce Erk activation in vivo upon illumination.

Of course, the Ras-Raf-MAPK pathway leading to pERK activation is only one of the pathways activated downstream of FGFR. Akt and PLC-γ pathways are also crucial to different aspects of cell behaviour, such as regulating cell morphology, migration and survival^23^. Therefore, testing differences in pERK levels is capturing only a subset of the full potential of FGFR function (see “Discussion” for further details).

### Opto-htl can induce heart cells in a *heartless* mutant

To further test the functionality of Opto-htl, we expressed it in a null *htl* mutant background to explore the extent of rescue of phenotypes at different stages upon light illumination. Homozygous mutants for *htl* undergo improper spreading of mesoderm cells at stage 10 forming irregular and multilayer arrangements^21^. They subsequently lack pericardial precursors, a proper muscle structure, pattern and, as the name suggests, fail to form a proper heart^24^. A previous tool to induce *htl* hyper activity, htl-λ^31,34^ was able to partially rescue the *htl* mutant, with some Eve-positive cells (precursors for pericardial and dorsal muscles) in the mesoderm restored.

**Figure 2:**
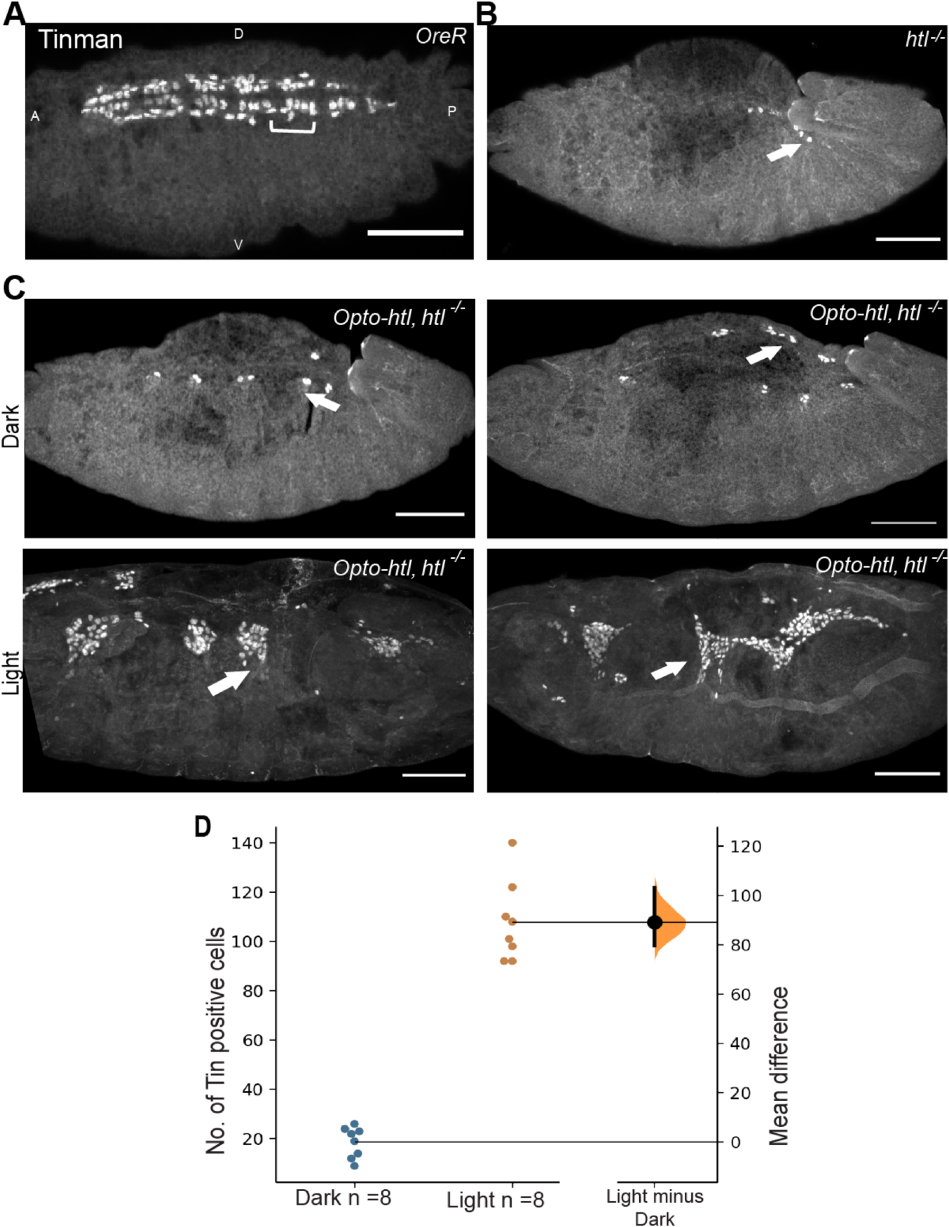
Rescue of *htl* mutant using Opto-htl. Tin positive heart cells in (**A**) wild-type and (**B**) *htl* homozygous mutant embryo. Tin is expressed in four cardioblasts per hemisegment (brackets) and in a subset of pericardial cells in OreR. Arrows show residual Tin-positive cells in a homozygous htl mutant embryo which fails to develop a heart. (**C**) *twi*::Gal4 > Opto-htl is expressed against *htl* null background and embryos illuminated and stained with Tin antibody. Two homozygous mutants are compared under dark and light conditions for Tin-positive cells. Arrows indicate Tin positive cells of the heart specified on the dorsal side of the embryos. (**D**) Quantification of the number of Tin-positive cells of the heart at stage 16 under dark and light conditions for embryos described in (**C**). Scale bar = 50 mm. A = Anterior, P = Posterior, D = Dorsal, V = Ventral view. In (**D**), the black bar on the right represents the 95% confidence interval with the Bootstrap distribution shown in orange^69^.

We tested for rescue of heart cells at stage 16 in homozygous *htl* mutants expressing *twi:*:Gal4 > Opto-htl with and without light activation. Tinman (Tin) is a transcription factor required for the specification of all heart cells and a subset of muscles^45^. At stage 15/16, Tin is expressed in a major portion of the cardioblasts and pericardial cells^46^. Tin staining is shown in OreR and *htl* null mutant embryos in Fig. 2A and B respectively. In OreR embryos, Tin is expressed in four out of six cardioblasts per hemisegments (brackets in Fig. 2A). *htl* null mutant embryos fail to express Tin positive cells (only a few residual cells observed) and hence do not form a heart. We found that *htl* null mutant embryos expressing Opto-htl maintained in light showed restoration of Tin-positive heart cells at stage 16 when compared to the same genotype embryos kept in the dark (Fig. 2C-D). In comparison, in wild-type embryos, there are around 104 Tin-positive cells in hemisegments A2–A8 (52 cardioblasts and pericardial cells each) at late stage 15/16^46^. Although Tin-positive cells were greatly increased under light activation of Opto-htl in *htl* null embryos, there was substantial variation in their spatial arrangement between embryos. The Tin-positive cells were located near the embryonic midline, but they failed to form a coherent heart structure. This is likely a consequence of embryos failing to undergo uniform mesoderm spreading over the ectoderm early on.

Opto-htl showed no rescue of phenotypes during early stages of development (Fig. S1). Since Opto-htl expression levels are generally low during early stages, it is possible that rescue during this stage requires higher levels of FGFR signalling than we currently achieve. These results are consistent with previous findings that Htl-dependent cell fate decisions in the mesoderm after stage 10 are decoupled from its role in mesoderm spreading^33^, though formation of robust organs requires synergy between both processes.

### Activation of Opto-htl induces ectopic Tin-positive cardioblasts

**Figure 3:**
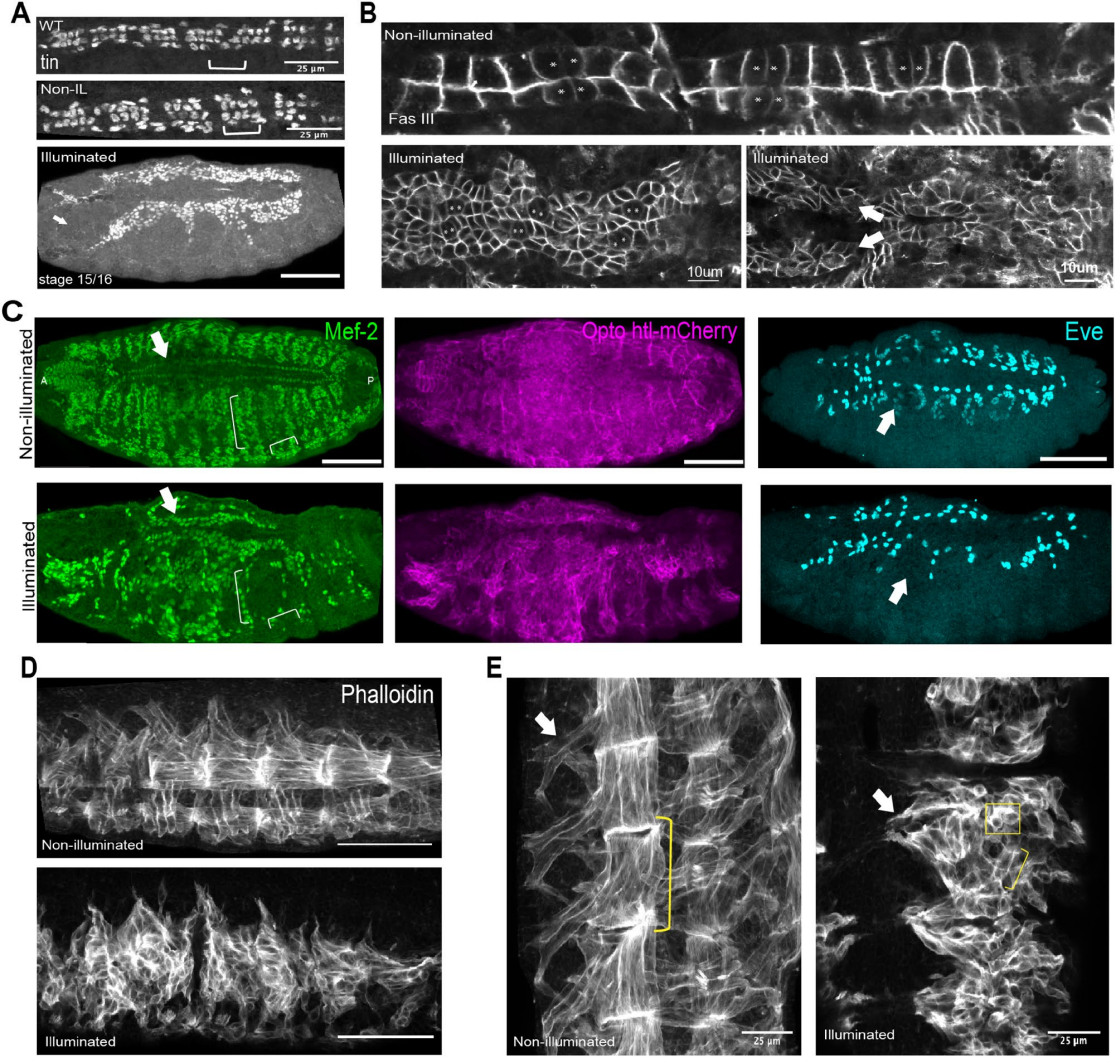
Mesoderm specific defects in *twi*::Gal4 > UAS—htl-CRY2-mCherry embryos kept under constant light. (**A**) Tinman staining pattern in illuminated wild-type (WT) embryos, *twi*::Gal4 > Opto-htl embryos kept under dark and illuminated conditions. (**B**) *twi*::Gal4 > Opto-htl embryos stained with Fas3 and imaged at stage 16 under dark conditions (top) and when illuminated by constant 488 nm light (bottom). Svp-positive cells, marked with an asterisk, are identified by lower Fas3 expression. Arrows denote branching of the heart structure. (**C**) *twi*::Gal4 > Opto-htl embryos stained with Mef-2 and Eve antibodies at stage 16 under dark and illumination conditions. Arrows correspond to phenotypes described in the text. (**D**) Muscle structure in *twi*::Gal4 > Opto-htl embryos kept under dark and light conditions visualised using Phalloidin staining. (**E**) As (**D**) with muscles imaged at higher magnification. Arrows point to extended Ventral Oblique muscles, brackets represent extended muscle fibres, square box shows unfused myoblasts. Scale bar = 50 μm unless stated otherwise. VM = Visceral mesoderm, CB = Cardioblasts, PC = Pericardial cells.

Light activation of Opto-htl in the mesoderm of wild-type embryos interfered with embryonic development, leading to a significant increase in lethality. We focused on the effect of Opto-htl activation on the development of mesoderm-derived tissues, particularly the heart. The heart in wild-type and Opto-htl embryos kept under dark is composed of two rows of cells (Fig. 3A; WT, Non-IL) with a repeated pattern of four Tin-positive and two Seven-up (Svp) positive cells per hemisegment^47,48^. The cells comprising these two rows are specified on both lateral sides of the embryo by the end of stage 12 after which they start migrating towards each other and match in a highly precise fashion^49,50^, (Fig. 3B top, Movie S4A–B). OreR embryos illuminated at 1 mW showed the regular four-two Tin expression pattern (Fig. 3A, WT) and hatched normally (Movie S5); confirming that our light exposure protocol itself does not induce any phenotypic defects.

Upon illumination, the stage 16 heart in *twi*::Gal4 > Opto-htl embryos showed a significant increase in the number of cardioblasts (Movie S6A–C, Fig. 3A–B Illuminated). There was an increase in the number of cardioblasts per hemisegment, but with substantial variation between embryos (Table 1). The clusters still had Svp-positive cells appearing in doublets, identifiable by reduced Fasciclin-III (Fas3) expression and morphology^50^ (asterisk in Fig. 3B) as in wild-type embryos, but they no longer matched with their contralateral partners. In some cases, the heart structure even became discontinuous (Movie S6A,C). *twi*::Gal4 > *hand*::GFP;Opto-htl embryos kept in dark showed heart cell migration and matching similar to wild-type (Movie S7). *hand*::GFP is expressed in these embryos from stage 13 onwards and expression is maintained in all the heart cells and the adjacent visceral mesoderm throughout embryogenesis. The observed asymmetries in the light-activated Opto-htl embryos suggested that the ectopic heart cells were generated asymmetrically between the contralateral sides of the embryos. Some embryos even developed multiple branches emanating from what appeared to be the primary heart vessel (arrowhead Fig. 3B). Therefore, we see that Opto-htl activation can significantly impair not only the number of heart cells but also the overall structural organisation of the dorsal vessel.

**Table 1:** Number of Mef2-positive cardioblasts, Eve-positive pericardial cells and DA1 muscles in OreR, *twi*::Gal4 > Opto-htl (dark) and *twi*::Gal4 > Opto-htl (illuminated) embryos. Cells were counted at stage 15/16 from hemisegments A3-A4.

Condition/genotype	Mef2-CB	Eve-PC	DA1
OreR (n = 12)	12	4	2
Dark-twi > Opto-htl (n = 10)	12	4	2
Light-twi > Opto-htl (n = 10)	29	6	0
	32	7	0
	41	9	0
	26	3	0
	20	6	0
	31	7	0
	30	6	0
	27	7	0
	21	3	0
	23	4	0

### Activation of Opto-htl disrupts mesoderm-derived muscle formation

Htl is crucial for the specification of founders of somatic muscles in the developing embryo^33,34^. Therefore, we predicted that light activation of Opto-htl embryos would disrupt embryonic muscle formation. In wild-type embryos, a cluster of Eve-positive cells around both sides of the heart marks the future (DA1) dorsal muscles (Fig. 3C top right)^51,52^. Mef-2 is a key transcription factor that marks and directs proper specification of the heart and body wall muscles^53,54^. In *htl* null mutants, there is a lack of Eve-positive precursors in the mesoderm and Mef-2 expression in mesoderm-derived tissues is significantly reduced^21^. Given the role of Htl signalling in specification of both Eve and Mef-2 expressing cells during the patterning of the mesoderm, we used antibodies against these markers to study how their expression is affected upon Opto-htl activation. Further, we stained for phalloidin to visualise the pattern and arrangement of body wall muscles in late-stage embryos.

Upon staining with Eve antibody, we observed that the DA1 muscle precursors are completely missing from Opto-htl embryos kept under light (Fig. 3C, Eve). These embryos also showed a reduced number of muscle-forming Mef-2 positive cells (Fig. 3C, brackets). From our phalloidin staining of the muscle patterns at stage 16, we see that Opto-htl embryos kept in dark have regularly arranged somatic muscles with attachment to the body wall (Fig. 3D). This pattern was disrupted in Opto-htl embryos kept under light, with the muscles appearing diffused and disrupted (Fig. 3D,E, Illuminated). These embryos also lacked ventral oblique muscles (Fig. 3E arrowhead). They exhibit only very small muscle fibres (Fig. 3E brackets) as compared to the extended fibres observed in the non-illuminated embryos and a variable number of unfused myoblasts (Fig. 3E square) are seen. Illuminated embryos exhibit significant defects in not just myoblast specification but also extension and fusion. *htl* null mutants exhibit myotube guidance defects since Htl regulates F-actin localisation and levels in myotubes during muscle development^55^. Therefore, overactivation using Opto-htl appears to interfere with the regulation of cytoskeletal changes required for proper myotube guidance.

Overall, we see that activation of Opto-htl can disrupt muscle founder specification, fusion and guidance. There is a complete loss of structural organisation within the somatic muscles and these defects likely contribute towards the observed lethality in light-activated opto-Htl embryos.

### Opto-htl allows non-uniform activation of Htl signalling within the *Drosophila* embryo

Thus far, we have only used uniform illumination conditions so that Opto-htl is activated throughout the mesoderm. Next, we aimed to control the spatial activation of Opto-htl to create heterogenous regions of Opto-htl activity within an embryo. This is particularly challenging as the target tissues lie deep within the embryo, not at the cell surface. Therefore, it is difficult to achieve the level of activation precision seen with optogenetic approaches in cell culture or the early embryo (when the cells are near the embryo surface)^42,56,57^. Here, we utilised light-sheet microscopy to generate a spatially restricted region of Opto-htl activation within a developing embryo. Since the light-sheet illuminates and collects the signal from a single optical section at a time, we are able to partition a given embryo into illuminated and non-illuminated sections^38^.

**Figure 4:**
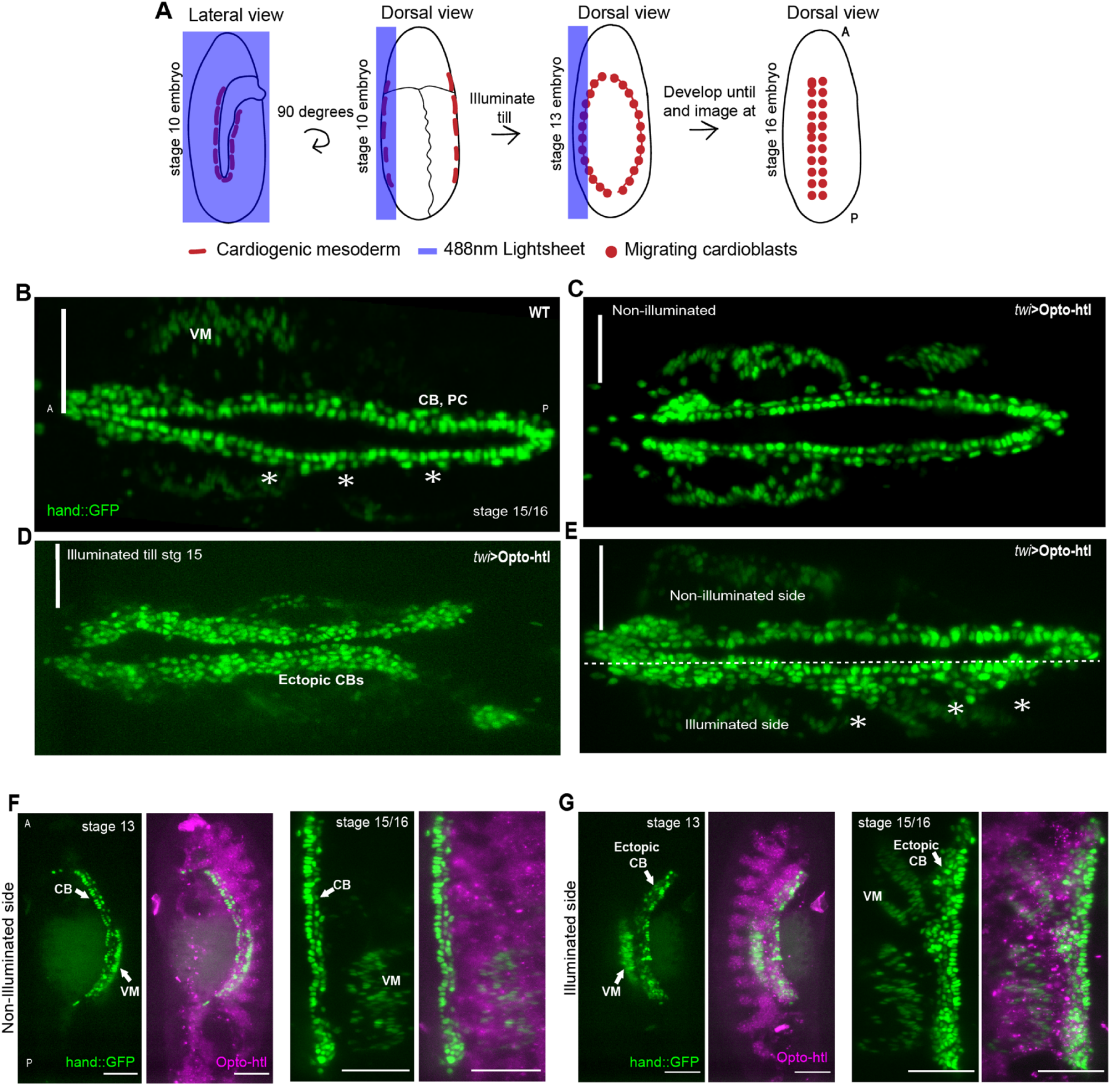
Spatial control of Opto-htl activation. (**A**) Schematic of the imaging protocol for half-illumination of embryos on a light-sheet microscope. (**B**) Dorsal view of a control embryo expressing *hand*::GFP. (**C**) *twi*::Gal4 > *hand*::GFP;Opto-htl embryo kept in dark and imaged at stage 15. (**D**) *twi*::Gal4 > *hand*::GFP;Opto-htl embryo fully illuminated from stage 5 till stage 15. (**E**) Dorsal view of a *twi*::Gal4 > *hand*::GFP;Opto-htl embryo illuminated as described in (**A**) and imaged at stage 16. (**F**) Non-illuminated and (**G**) illuminated sides of a *twi*::Gal4 > *hand*::GFP;Opto-htl embryo. Left panels, stage 13. Right panels, stage 15/16. VM = Visceral mesoderm, CB = Cardioblasts, PC = Pericardial cells. Scale bar = 50 mm.

We mounted *twi*::Gal4 > *hand*::GFP; Opto-htl embryos such that the illumination plane was parallel to the long-axis of the embryo. These embryos express GFP at stage 16 in all the heart cells and the adjacent visceral mesoderm. To generate spatially heterogeneous activation of Opto-htl, we scanned the Opto-htl embryos with the 488 nm laser to a depth of ~ 35um from the embryo surface on one lateral side, to illuminate the heart precursors on only one side of the embryo (Fig. 4A). At stage 16, we imaged the embryo from the dorsal side to image the heart and compared *hand*::GFP signals from the illuminated and non-illuminated sides. In embryos lacking the Opto-htl construct (*hand*::GFP control), the light sheet illumination did not alter the heart structure at stage 16 and these embryos developed normally (Fig. 4B, Movie S4A–B). In contrast, uniformly illuminating Opto-htl expressing embryos with a similar light intensity resulted in a significant increase in the number of cardioblasts compared with both *hand*::GFP embryos and *hand*::GFP, twi > Opto-htl embryos kept in the dark (Fig. 4C,D).

When we asymmetrically illuminated Opto-htl expressing embryos we observed a striking difference between the number of heart cells on both sides (Fig. 4E). The non-illuminated side resembled the dark condition phenotype (Fig. 4B), while the illuminated side showed multiple ectopic cardioblasts (Fig. 4E, asterisk). Therefore, we can spatially define distinct domains of Htl over-activation deep within the developing embryo.

We observed ectopic cardioblasts in the illuminated side immediately after the end of illumination at stage 13 (Fig. 4F,G, left). By the end of stage 15 (Fig. 4F), the non-illuminated side resembled wild-type embryos in terms of its arrangement and number of heart cells. Meanwhile, the illuminated side had substantial ectopic cardioblasts arranged irregularly along the embryonic midline (Fig. 4G). These results suggest that the two lateral sides of the mesoderm develop independently from each other, and they cannot correct for defects in the formation of their contralateral partner. This is consistent with the fact that the mesodermal cells do not mix extensively along the lateromedial axis and their spatial information with respect to one another is largely conserved during their migration.

### Severity of Opto-htl induced phenotypes is dosage dependent

**Figure 5:**
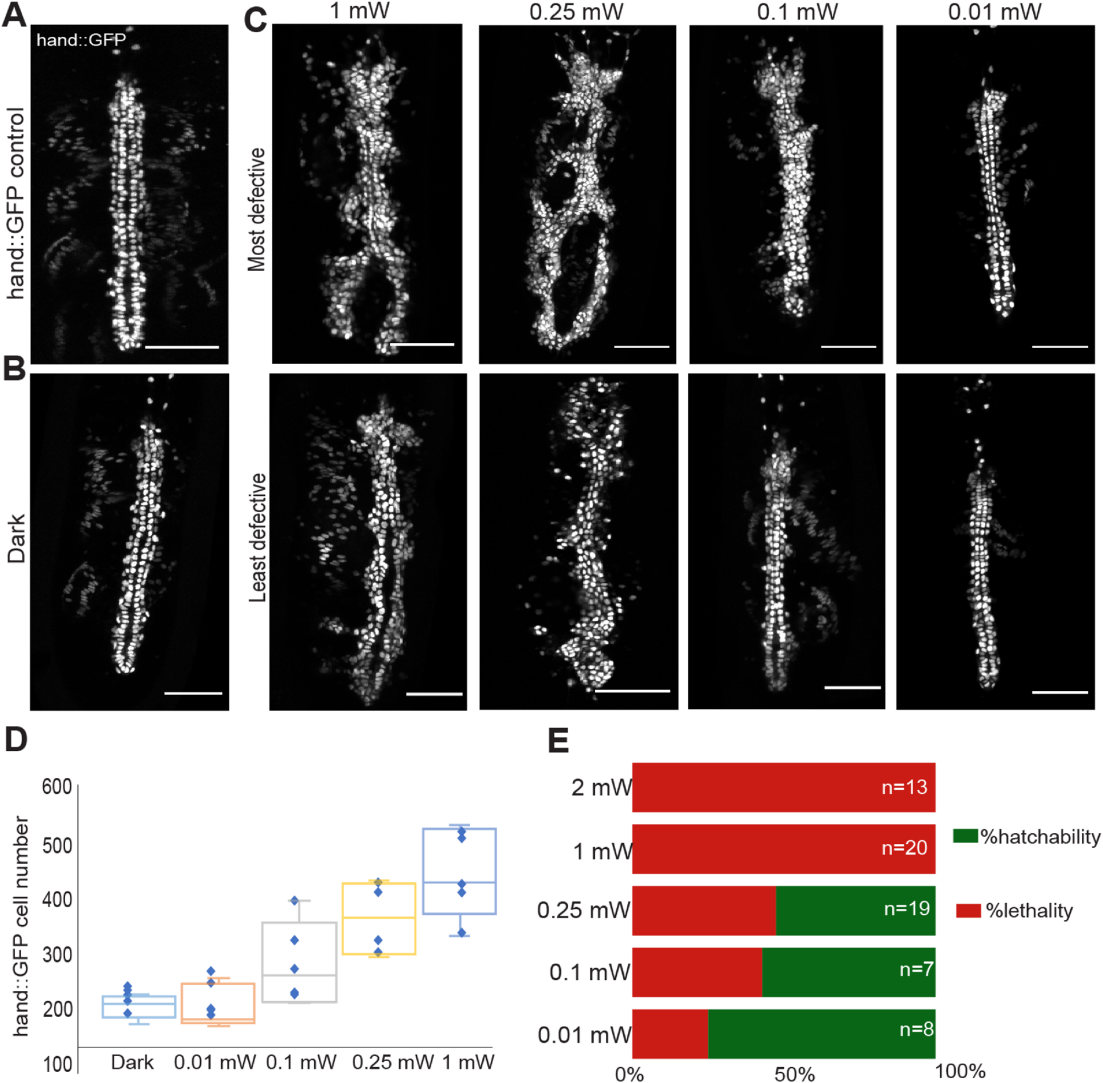
Opto-htl induced phenotype depends on light intensity. (**A**) Heart at stage 16 in a *hand*::GFP control embryo. (**B**) Heart at stage 16 in a *twi*::Gal4 > *hand*::GFP;Opto-htl embryo kept in the dark throughout development. (**C**) Representative images of the heart at stage 16 in *twi*::Gal4 > *hand*::GFP;Opto-htl embryos illuminated from stage 5 till stage 16, at different light intensities. (**D**) Quantification of *hand*:GFP cell number for embryos illuminated at different intensities (n = 5 for each condition). The top and bottom of the box represent the third quartile and the first quartile, respectively. The median divides the box. The whiskers are error bars: upward from the third quartile to the maximum, and downward from the first quartile to the minimum. (**E**) Survival rate of *twi*::Gal4 > Opto-htl embryos when illuminated at different intensities throughout development. Scale bar = 50 μm.

We observed the aforementioned phenotypic defects in Opto-htl embryos upon continuous and uniform illumination at a fixed intensity of 1 mW (measured at the sample plane). Next, we explored how the phenotype varies in response to changes in the light intensity used for illumination. We varied the dosage of signalling by illuminating Opto-htl embryos with different light intensities using a LED light base as the light source. We used light intensities at 2 mW, 1 mW, 0.25 mW, 0.1 mW, and 0.01 mW (measured using an intensity power meter set at the 488 nm range) for illuminating *twi*::Gal4 > *hand*::GFP; Opto-htl embryos. Embryos were illuminated continuously from stage 5 up until stage 15/16 (~ 12 h) and then the heart in each embryo was imaged. As a reference, we show the *hand*::GFP pattern in a *hand*::GFP expressing embryo without Opto-htl (Fig. 5A) and an Opto-htl expressing embryo kept in the dark (Fig. 5B). In Fig. 5C we depict the most and least defective heart images of Opto-htl expressing embryos at each light activation intensity, based on the number of ectopic cardioblasts. At low intensities (0.01 mW), the least defective embryos closely resembled *hand*::GFP control and embryos maintained in the dark condition (Fig. 5A,B), with only a few ectopic cardioblasts. At 0.1 mW, ectopic cardioblasts were more apparent in some embryos, though the overall structure still resembled the non-illuminated embryos. For intensities 0.25 mW and above, the heart defects were marked, with major structural changes to the heart vessel (branching, inconsistent matching). *hand*::GFP cell number increases rapidly for light activation intensity above 0.01 mW, as quantified in Fig. 5D.

After imaging the embryos at stage 16, we let them develop further and scored their hatching rate. We found that increased intensity led to lower survival rates. At 0.1 mW exposure, we observe a survival rate of around 57% as compared to a 100% lethality observed at intensity values of 1 mW or higher (Fig. 5E). Despite significant ectopic cardioblasts, 52% of the embryos illuminated at 0.25 mW still managed to hatch, suggesting that the embryos can tolerate a large increase in cardioblast number at this stage. However, we did not track these embryos throughout development after hatching so their survival through larval stages is unknown.

We compared these results with the other constitutively active form, htl-λ^31,34^ htl-λ did not induce lethal phenotypic defects when expressed in the mesoderm. Tin and Eve patterns at stage 15 in *twi*::Gal4 > htl-λ embryos showed only small phenotypic variation from wild-type patterns (Fig. S3). The heart phenotype observed for htl-λ resembled that of Opto-htl embryos at lower intensity illuminations (< 0.1 mW). Of course, differences between the scales of action for the two constructs cannot be ruled out. At higher light activation, we see stronger phenotypes in the Opto-htl embryos as compared to htl-λ. Our results suggest that specification of the cardiac vessel within the mesoderm is sensitive to FGFR activity levels.

### Embryo development is sensitive to Opto-htl activation during stages 10–12

In the early embryo, Htl plays an important role in regulating mesoderm spreading^31,58^, but the dynamics of its action later in development are less well known. We next used Opto-htl to activate Htl signalling during distinct developmental time windows to try to disentangle its early and later effects on embryo development and to test when the developing mesoderm is most sensitive to Htl over-activation.

**Figure 6:**
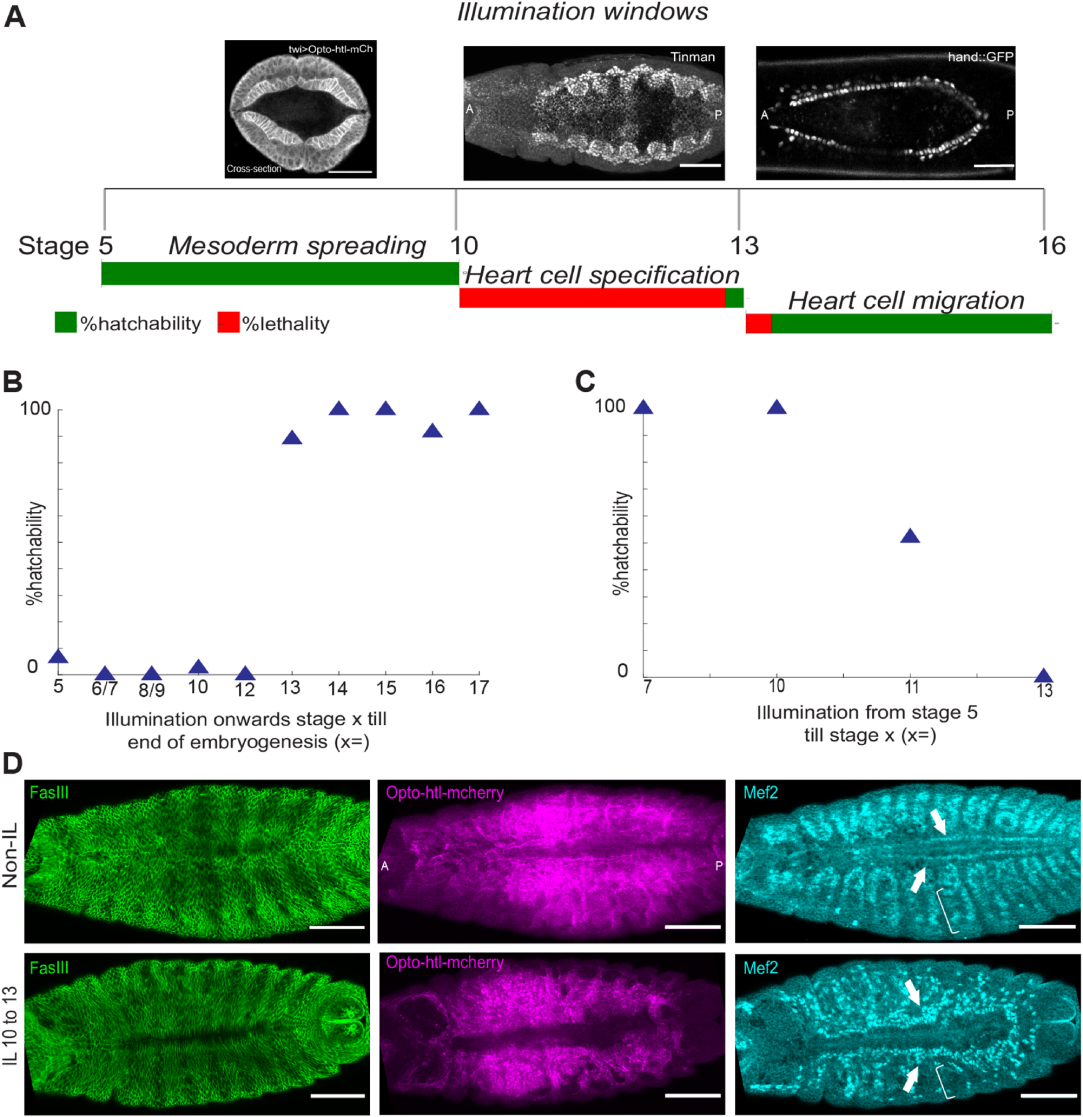
Temporal control of Opto-htl activation during development. (**A**) Hatching rate assay for *twi*::Gal4 > Opto-htl embryos illuminated during different time windows during development. (Top) Representative images of the embryo stage being illuminated. (Bottom) Hatching percentage, with the length of each bar representing 100%. (**B**) Hatching rate of *twi*::Gal4 > Opto-htl embryos when illuminated from the specified stage on the x-axis to the end of embryogenesis (**C**) Hatching rate of *twi*::Gal4 > Opto-htl embryos when illuminated from stage 5 until the specified stage on the x-axis (see Tables 2, 3 for embryo count) (**D**) *twi*::Gal4 > Opto-htl embryos stained with Fas3, Mef2 and mCherry antibodies under different illumination conditions. Arrowheads point towards Mef-2 marked cardioblasts and brackets represent loss of mef-2 positive muscle precursors. Scale bars = 50 μm.

We illuminated *twi*::Gal4 > Opto-htl embryos during three separate time windows (Fig. 6A): (1) from stage 5 to stage 10 (~ 2.5 h), during which the presumptive mesoderm forms the ventral furrow and undergoes spreading over the ectoderm to form a monolayer; (2) from late stage 10 to late stage 12 (~ 4 h), during which the specification of the presumptive mesoderm into different cell types occur; and (3) from stage 13 up to stage 16 (~ 4 h), during which cardioblasts migrate to the embryo midline and form the heart. We observe that nearly all embryos illuminated during windows (1) and (3) hatch. However, almost all embryos illuminated between stages 10 and 12 failed to hatch (Fig. 6A). As previously stated, Opto-htl levels at early stages—i.e. during time window (1)—can only be detected by anti-mCherry antibody staining, suggesting low expression levels (Movie S1).

We next performed illuminations during more fine-tuned developmental windows (Fig. 6B,C, Tables 2, 3). We see a sharp transition in survivability around stage 12. Embryos illuminated prior to stage 12 show high lethality, and embryos illuminated only after stage 12 show high survivability (Fig. 6B). Performing the opposite light illumination protocol, all embryos illuminated until stage 10 but then kept in the dark hatched. Embryos illuminated until stage 11 had a survivability of around 50%. Therefore, we conclude that developmental processes around stage 11–12 appear to be especially sensitive to Opto-htl activation when it is driven by *twi*::Gal4.

**Table 2:** Hatching rate of *twi*::Gal4 > Opto-htl embryos illuminated from specified stages until end of embryogenesis.

Illumination onwards stage x till hatching (stage x =)	Illumination time from cellularization stage	Stage Hallmark	No. of embryos screened	No. of embryos hatched	% hatchability
5	0 min	Cellularisation	31	2	6.4
6/7	40 min	Gastrulation	7	0	0
8/9	1–2:10 h	Germ band Elongation starts	5	0	0
10	2:10 h	Germ band Elongation finishes	40	1	2.5
12	5:10 h	Germ Band retraction starts	34	0	0
13	7:10 h	Midgut primordium forms a sack	19	17	89
14	8:10 h	Head Involution and DC starts	11	11	100
15	9:10 h	Dorsal Closure	18	18	100
16	11 h	Gut constrictions appear	12	11	91.6
17	14 h	First movements start; Trachea filling	16	16	100

**Table 3:** Hatching rate of *twi*::Gal4 > Opto-htl embryos illuminated during different time windows. (stg = stage).

Illumination window	Illumination duration	No. of embryos screened	No. of embryos hatched	% hatchability
Stg 5 till end of stg 7	40 min	7	7	100
Stg 5 till end of stg 10	2.5 h	7	7	100
Stg 5 till end of stg 11	5 h	17	9	52
Stg 5 till end of stg13	8 h	24	0	0

Interestingly, the specification of different cardiac progenitors is known to occur during stages 10–12^49^. To determine what defects were induced by illumination of Opto-htl during time window (2), we let embryos illuminated during this window continue to develop in the dark from stage 13 onwards and fixed them at stage 16. We observed that these embryos had ectopic Mef-2 positive cardioblasts (Fig. 6D, arrows), similar to Opto-htl embryos illuminated throughout development. Further, the Mef-2 positive muscle cells were also reduced as seen in the body wall muscles (Fig. 6D, brackets).

Activation of Opto-htl during the developmental period from late stage 10 until late stage 12 was both necessary and sufficient to induce the described mesodermal defects. This window is crucial for heart morphogenesis and requires precise levels of FGFR activation to ensure proper cell fate specification and robust development^49^. In contrast, later developmental processes (post stage 13) appear to be able to tolerate a much higher range of Htl activity, possibly because cell-fate specification has already happened by this time and/or cells can buffer any increased Htl activity.

## Discussion

We have demonstrated the use of an optogenetic tool (Opto-htl) to study spatiotemporal regulation of Htl signalling in vivo by controlling its level of activation within defined spatial and temporal windows. We found that the embryo is sensitive to Htl over-activation during the time window from late stage 10 till end of stage 12 (early stages of heart morphogenesis). Illumination from stage 13 onwards did not result in any remarkable defects (Fig. 6). Within the stage 10 to 12 window, the severity of the induced phenotype increased with increasing activation light intensity (Fig. 5C). Opto-htl provides a distinct advantage over previous studies of Htl over-activation, as it allows careful tuning of the strength of the pathway response at any point in development.

Htl plays a crucial role in shaping the mesoderm by ensuring (1) uniform spreading of the mesoderm over the ectoderm and (2) subsequent fate specification of different tissue precursors^21,22,33,59^. The earlier role of Htl in uniform spreading is independent of—though crucial for—fate specification and cell arrangement at later stages. This likely explains the heterogeneous pattern of restored Tin-positive cardioblasts in *htl* null mutants upon Opto-htl activation (Fig. 2C). Opto-htl can activate *tin* expression but is unable to correct the defective spreading in these embryos at early stages. Further, Opto-htl activation does not cause any expansion of mesodermal cells at these stages when expressed against a wild-type background in the mesoderm using *twi*::Gal4 (Fig. S2).

Opto-htl offers a greater exploration range for the role of Htl activity in controlling mesoderm cell behaviour than possible with other constitutively active receptors. In *twi*::Gal4 > Opto-htl embryos, Eve-positive DA1 muscles at stage 16 are completely missing from light-activated (1 mW) embryos at stage 16 (Fig. 3C). However, at low light activation, Opto-htl phenotype was similar to that of htl-λ with respect to the number of ectopic Tin-positive heart cells observed at stage 16 (Fig. S3, asterisk). It is possible that at higher light intensity of 1 mW, Opto-htl over-activation of the FGFR pathway is inducing conversion from a muscle cell fate to a cardiac or pericardial cell fate; *i.e.* Mef-2 positive muscle founders adopt a Tin-positive cardioblast fate and Eve-positive dorsal muscle cells adopt a pericardial fate. Therefore, Opto-htl potentially enables tuning of FGFR-dependent cell fate decisions within the developing mesoderm.

MAPK signalling dosage is critical in regulating cell proliferation and cell fate specification events in the mesoderm during stages 10–12 and it has previously been implicated to play a role in the development of several different cancer types^60^. We compared the developmental effects of constitutive activation at the receptor level with Opto-htl (potentially activating all downstream pathways) to activation of only the Ras/Raf/MAPK pathway in the mesoderm by driving Opto-SOS^40^ with *twi*::Gal4. Upon illumination, we observed similar phenotypes in the heart as with Opto-htl; abundance of Tin-positive cardioblasts and loss of DA1 muscles (Fig. S4). The phenotypes are pronounced at 4.5 mW illumination intensity, at which no DA1 muscles are observed and there is an abundance of Eve-positive pericardial cells and Tin-positive cardioblasts. At lower intensities, some of the DA1 structure is maintained. Hyperactivation of the Ras/Raf/MAPK pathway alone leads to phenotypes similar to Opto-htl in the mesoderm. However we cannot rule out contributions from other branches downstream of Htl; *i.e.* PI3K and PLC pathways^23^. Unfortunately, antibody staining for these pathways was ineffective and dynamic reporters that work well within the deep tissues of *Drosophila* are not currently available.

Our results suggest that Htl-dependent cell fate decisions are stage specific and dosage dependent. An increase in dosage leads to variations in different cell types of the heart and muscle lineage. These events are restricted to a time window for Htl over-activation between late stage 10 and late stage 12, though recall that the lower time bound is uncertain due to relatively lower expression levels of *twi*::Gal4 > Opto-htl at early stages. Using Opto-htl to control several parameters of FGFR activation—dosage, spatial region of activation, and timing of activation—we have provided insight into different modes of Htl action in the developing mesoderm. Achieving finer temporal scales is currently challenging due to lack of good biosensors of Erk and other pathways for live imaging in *Drosophila,* especially in deeper lying tissues. In future work, we aim to fine tune these parameters, to study how they affect cell fate changes *in htl* null embryos. We are currently developing live reporters for cardioblasts with clearer expression starting at earlier stages of heart development to explore fate specification dynamics and unravel whether the observed changes in cell number are related to over-proliferation of Tin-positive cardioblasts or switches in cell identity of other mesodermal cells.

FGFR signalling plays a crucial role in the development and maintenance of several different organ systems in humans (for example the heart, lungs, brain and skeletal muscles) and is also a target in disease therapies^61^. There is also evidence from animal studies that FGFR signal activation has potential for use in tissue regeneration and repair^62–64^. Optogenetic control over signalling pathways allows us to fine tune their activation or deletion in a highly controlled spatiotemporal manner. Studying the conditional effects of these manipulations on development and disease pathogenesis has the potential to lead to novel therapeutics.

## Materials and methods

### Fly stocks and genetics

UAS-htl-Cry2mCherry construct was generated using Gibson assembly (NEB) of four fragments: pPW vector (Drosophila Genomics Resource Center, Bloomington, IN) backbone digested and purified, synthesized src42A myristoylation (myr) signal sequence^65^, PCR amplified cytoplasmic region of *htl/btl* from freshly prepared cDNA (*Drosophila melanogaster*), PCR amplified CRY2mCherry sequence from the AddGene plasmid #26,866. The recombinant plasmid was sent to BestGene Inc for P-element transformation. *twi*::Gal4^66^ (FlyBase^67^ ID: FBti0002997, FBti0002998) was used to drive expression in the mesoderm and *byn*::Gal4 > UAS-myr-GFP was used to drive expression in the hindgut (kindly provided by Kenji Matsuno). To mark the heart cells for live imaging, we used *hand*::GFP (kindly provided by Zhe Han; GFP driven by the *hand* cardiac and hematopoietic (HCH) enhancer) and formed a stable line: *hand*::GFP;UAS-htl-Cry2mCherry.

For the rescue experiments, UAS-htl-Cry2mCherry on 2nd chromosome was combined with the *htl*^*AB42*^ null mutant^25^ (FlyBase^67^ ID: FBal0057264) on 3rd chromosome. Similarly, *twi*::Gal4 on 2nd chromosome was combined with *htl*^*AB42*^ null mutant to form a stable line. UAS-htl-Cry2mCherry; htl^AB42^/TM3-ftz-lacz virgin females were crossed to *twi*::Gal4; htl^AB42^/TM3-ftz males resulting in 25% homozygous mutant embryos expressing *twi*::Gal4 > UAS-htl-Cry2mCherry. All fly lines were raised at 25 °C.

### Identification of homozygous *htl* mutant embryos for rescue experiments

For the rescue crosses, a balancer chromosome carrying a lacZ-transgene was used. lacZ expression was driven by the ftz-promoter and was detected in embryos using anti- β-Gal antibody (DSHB 40-1a). For cross-section staining, lacZ-positive and lacZ-negative embryos were sorted under a dissecting microscope based on the β-gal staining pattern prior to cutting in 70% glycerol using a 25-gauge needle.

### Immunostaining

Embryos were collected at the desired stages, then dechorionated using bleach and fixed in heptane saturated with 37% formaldehyde (40 ml of heptane with 40 ml of 37% formaldehyde) for 50 min. The vitelline membrane was subsequently removed using a needle. Prior to immunostaining, the embryos were blocked in 10% BSA-PBS. Antibodies used were mouse anti- β-Gal (1:100, DSHB), rabbit anti-mCherry (1:100, Abcam), guinea pig anti-Eve (1:800, kindly provided by James Sharpe), rabbit anti-dpERK (1:100, CST), mouse anti-Fas3 (1:300, DSHB), Rabbit anti-Tin (1:1000, kindly provided by Manfred Frasch), mouse anti-myosin heavy chain (1:100, DSHB), rabbit anti-Mef2 (1:800, kindly provided by Eileen Furlong). Primary antibodies were detected with Alexa Fluor-labelled secondary antibodies (1:500; LifeTech). Embryos were mounted in AquaMount (PolySciences, Inc.) and imaged using a Zeiss LSM710 microscope with a C-Apochromat 40 × water-immersion objective or a Nikon A1-RS scanning confocal microscope with 40 × water-immersion objective. For dpERK intensity comparisons, both dark and light condition embryos were collected, stained and imaged under same conditions (antibody dilutions, laser power, etc.).

### Illumination experiments

To score their hatching rate, embryos were imaged on a bright-field stereomicroscope at 25 °C. To maintain the dark condition where needed, embryos were observed with the light source covered with amber paper to block 488 nm light. For illuminating embryos, we used a Nikon LED light base as the light source and measured different light intensities using an intensity power meter set at the 488 nm range. All experiments were carried out at 1 mW (measured at the sample plane) unless otherwise stated. For different time-window illuminations, stage 5 embryos were collected in the dark condition (488 nm light blocked by amber paper) and then exposed to light. For keeping embryos in dark for recovery after illumination, we used a box covered with aluminium foil. For staining experiments, dark and light condition embryos were fixed and stained simultaneously at exactly similar stages. All embryos were collected and staged according to Ortega and Hartenstein^68^.

### Light-sheet microscopy for spatially controlled activation

Embryos were dechorionated using bleach, mounted into a capillary containing 1% low-melting agarose (Sigma) in an upright position and imaged on a Z.1 light-sheet fluorescence microscope (Carl Zeiss, Germany) using a 40 × water immersion objective. The light-sheet microscope was equipped with 30 mW 488 nm and 20 mW 561 nm lasers with BP505–545 and LP585 emission filters respectively. Embryos were imaged using dual-side illumination by a light-sheet modulated into a pivot scan mode. The 488 nm excitation laser was used at 6% power with 7.5 ms exposure time and the 561 nm excitation laser was used at 13% laser power with 20 ms exposure time. For control embryos, only the 561 nm laser was used for scanning.

For spatial control of activation, embryos were mounted vertically and scanned with the 488 nm laser from the lateral side such that the light-sheet was parallel to the plane of the embryo. ~ 35 um of slices from the embryo surface were scanned so as to illuminate the heart precursors on only one side of the embryo. We scanned embryos with the 488 nm laser from stage 10 till stage 13 and then we let embryos develop in the dark until stage 16 (no scanning with 488 nm laser). At stage 16, we scanned the dorsal side of the embryos to image the heart and compared signals from the illuminated and non-illuminated sides.

## Supplementary Information

Supplementary Fig. 1.

Supplementary Fig. 2.

Supplementary Fig. 3.

Supplementary Fig. 4.

Supplementary Video 1.

Supplementary Video 2.

Supplementary Video 3.

Supplementary Video 4.

Supplementary Video 5.

Supplementary Video 6.

Supplementary Video 7.

Supplementary Video 8.

Supplementary Video 9.

Supplementary Video 10.

Supplementary Information.
