# Supplementary figures and images for: Spatiotemporal sensitivity of mesoderm specification to FGFR signalling in the Drosophila embryo
Source: Sci Rep. 2021 Jul 8;11:14091. doi: 10.1038/s41598-021-93512-1 (PMC8266908; doi:10.1038/s41598-021-93512-1)

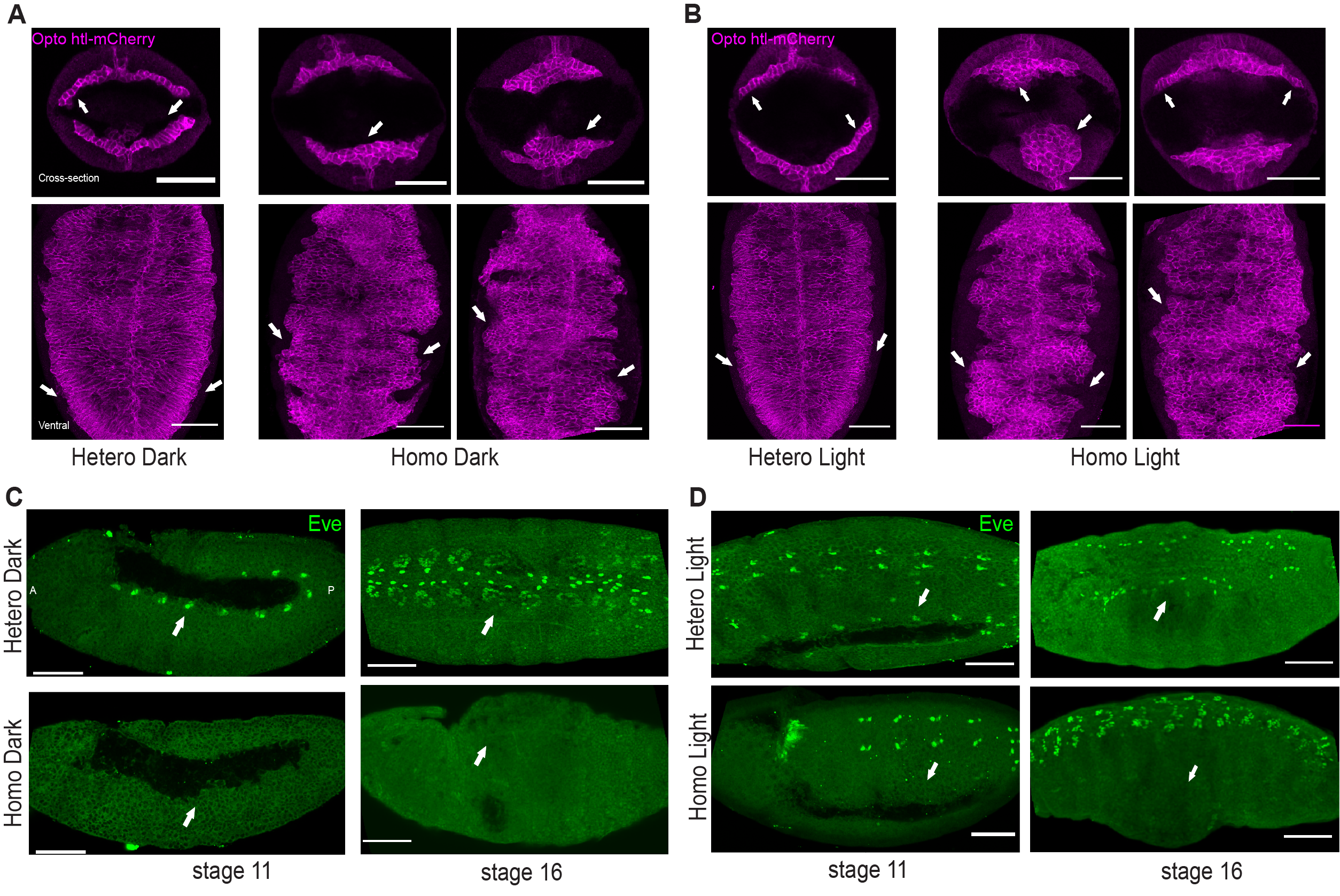

Supplement: Supplementary file 1 — Supplementary Fig. 1. [file 41598_2021_93512_MOESM1_ESM.png]

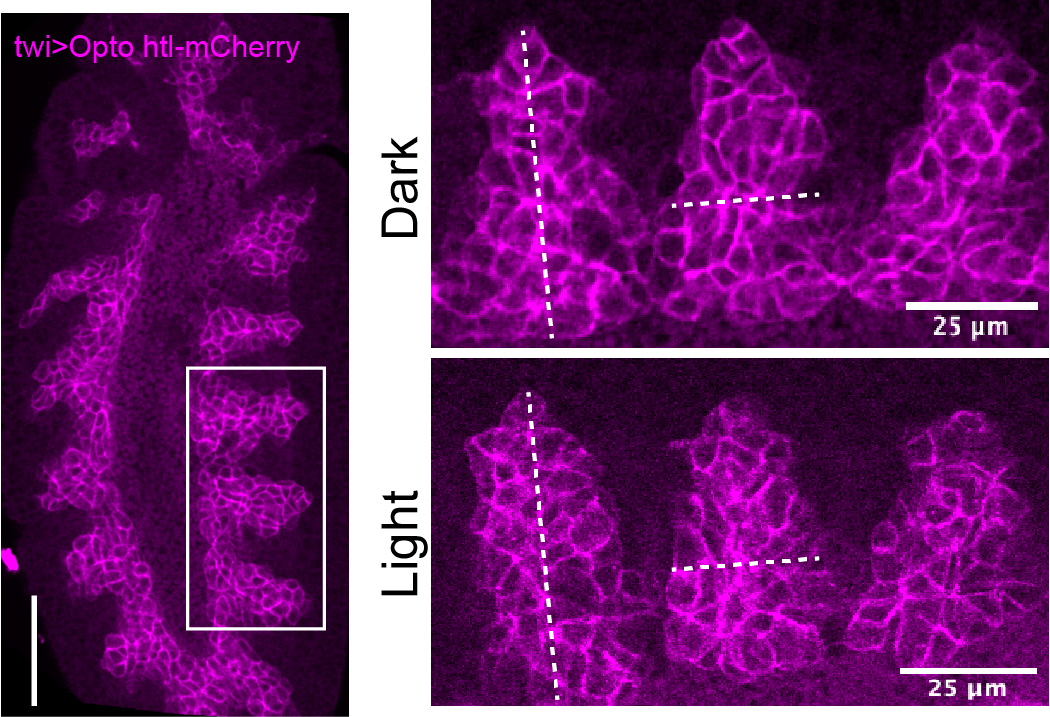

Supplement: Supplementary file 2 — Supplementary Fig. 2. [file 41598_2021_93512_MOESM2_ESM.png]

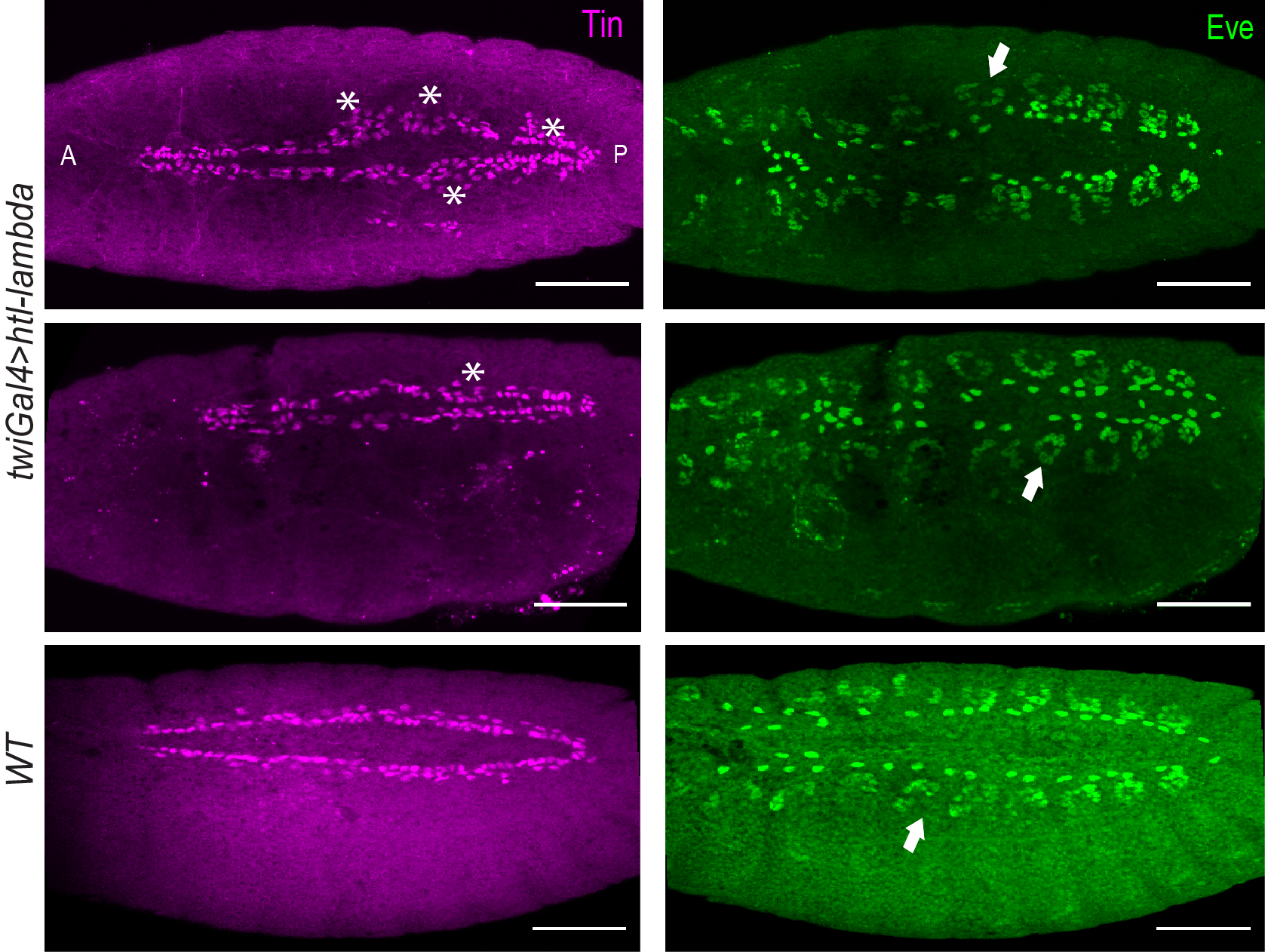

Supplement: Supplementary file 3 — Supplementary Fig. 3. [file 41598_2021_93512_MOESM3_ESM.png]

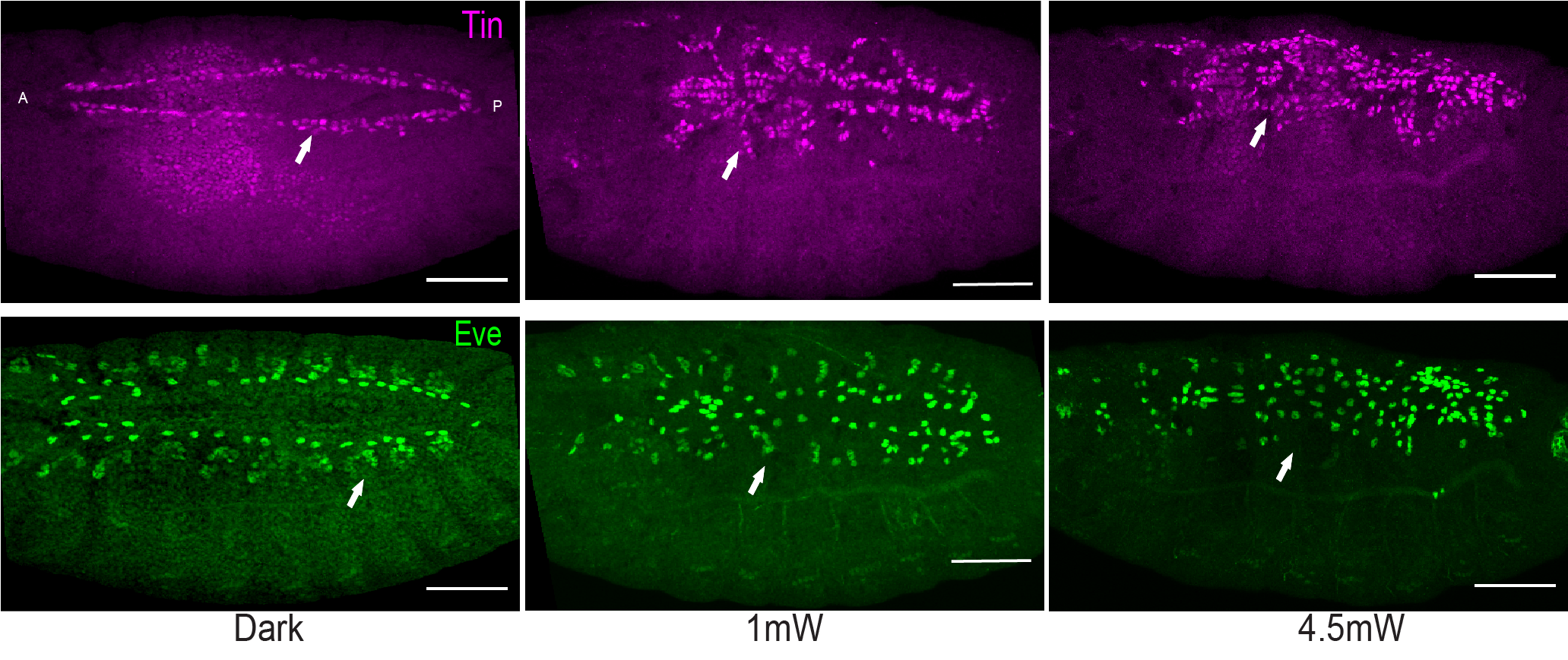

Supplement: Supplementary file 4 — Supplementary Fig. 4. [file 41598_2021_93512_MOESM4_ESM.png]
