# Supplementary material for: Spatiotemporal sensitivity of mesoderm specification to FGFR signalling in the Drosophila embryo
Source: Sci Rep. 2021 Jul 8;11:14091. doi: 10.1038/s41598-021-93512-1 (PMC8266908; doi:10.1038/s41598-021-93512-1)
Supplement: Supplementary file 15 — Supplementary Information. [file 41598_2021_93512_MOESM15_ESM.pdf]

Supplementary Materials for

Spatiotemporal sensitivity of mesoderm specification to FGFR signalling in the *Drosophila* embryo

V. Yadav[1], N. Tolwinski[2,3] and T. E. Saunders[1,3,4,5,*]

[1] Mechanobiology Institute, National University of Singapore, Singapore
[2] Yale-NUS, National University of Singapore, Singapore
[3] Department of Biological Sciences, National University of Singapore, Singapore
[4] Institute of Molecular and Cell Biology, A*Star, Singapore
[5] Warwick Medical School, University of Warwick, Coventry, United Kingdom
* For correspondence: dbsste@nus.edu.sg

This PDF file includes:

Fig. S1 to S4
Supplementary Movie S1-S7 Legends
Sequences and Primers

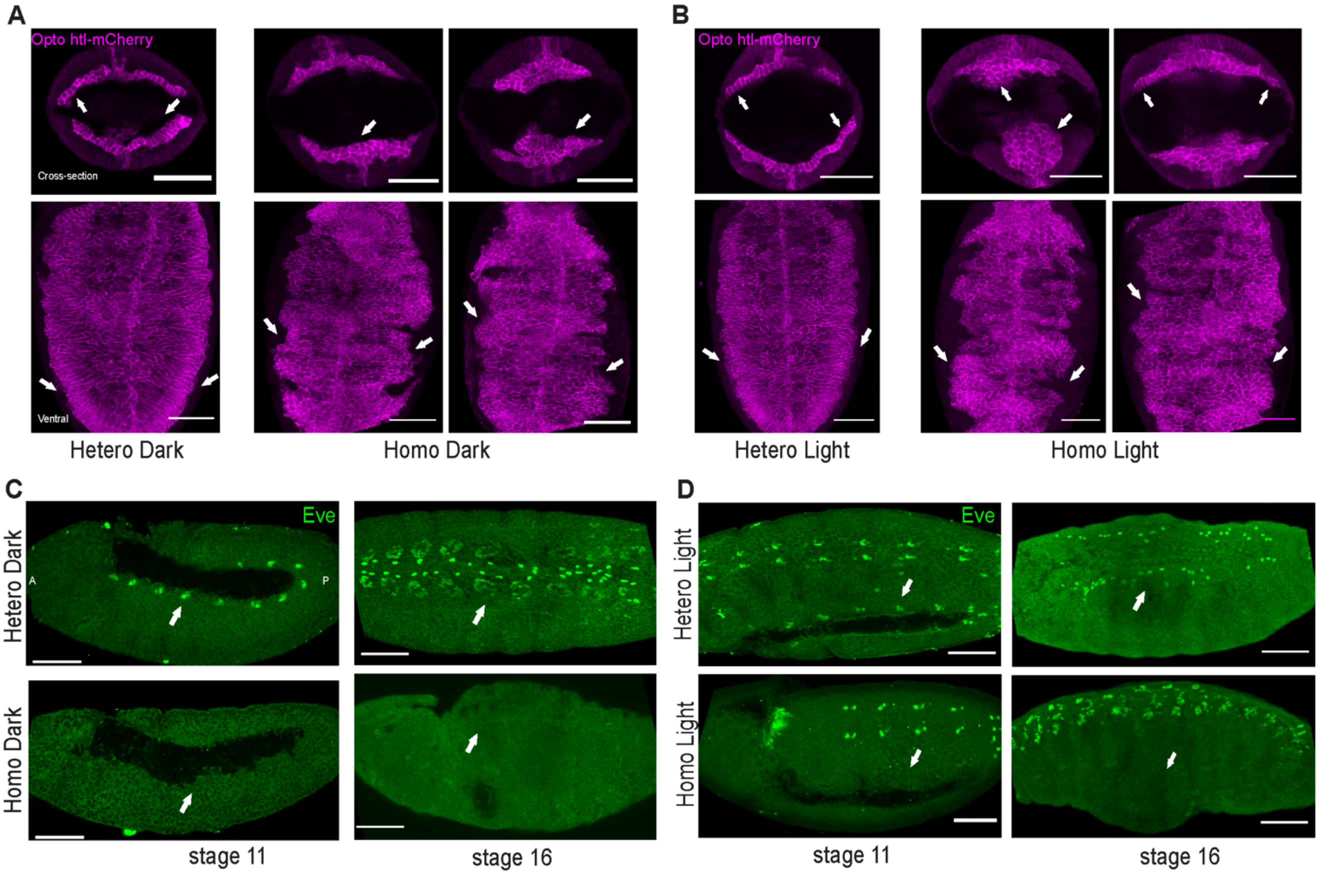

Supplementary Fig. 1 Rescue phenotypes using UAS-Opto-htl

A) Mesoderm spreading in heterozygous and homozygous *htl* mutant embryos kept under dark conditions, imaged at the cross-section (top row) and from the ventral side (bottom row). B) Mesoderm spreading in heterozygous and homozygous *htl* mutant embryos illuminated from stage 5 till stage 10. Upon illumination, homozygous mutants expressing Opto-htl exhibit clustering of mesoderm cells and non-uniform spreading with flattening observed occasionally in some regions. No rescue of Eve-positive cells is observed in the homozygous mutants under light either at stage 11 (C) or stage 16 (D). Scale bar = 50μm.

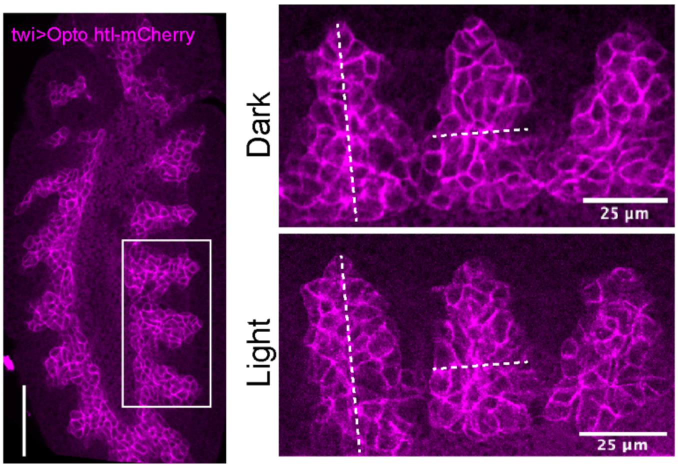

Supplementary Fig. 2 Mesodermal cells in Opto-htl embryos

twi::Gal4>Opto-htl-mCherry embryos fixed and stained at stage 11 with anti-mCherry antibody under dark and light conditions. No expansion of mesodermal cells is observed upon light activation. Scale bar = 50µm unless stated otherwise

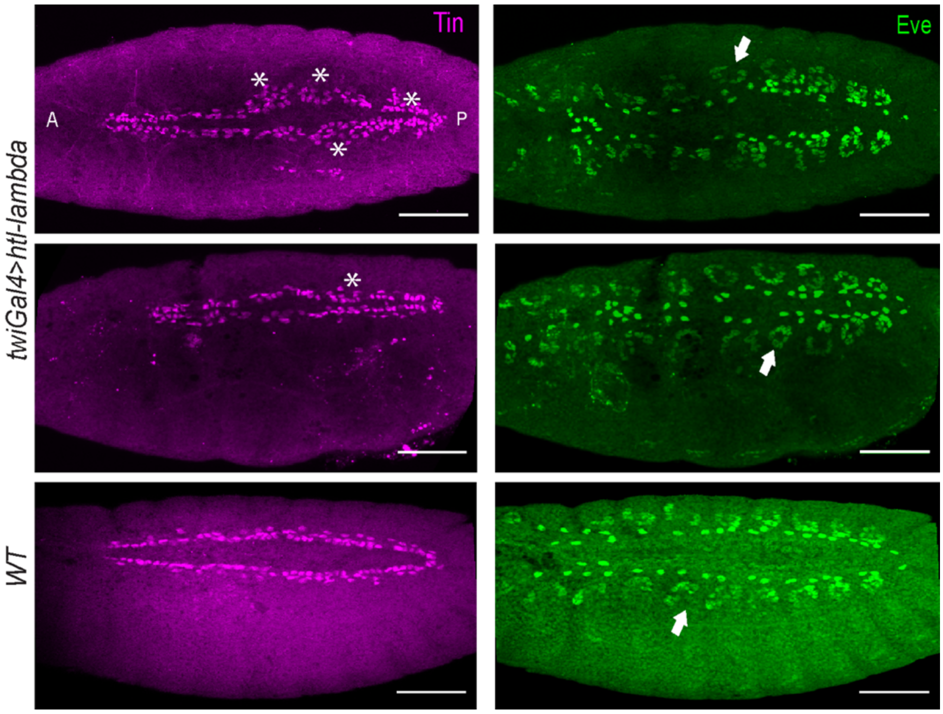

Supplementary Fig. 3 htl-λ activation in the mesoderm

Dorsal view of *twi::*Gal4>UAS-htl-λ embryos fixed and stained at stage 15 with Tin and Eve antibody, compared with WT embryos at the same stage. Asterisks represent ectopic Tin-positive cells. Arrowhead point towards eve-positive DA1 muscles and pericardial cells in a given hemi segment**.** Scale bar = 50µm.

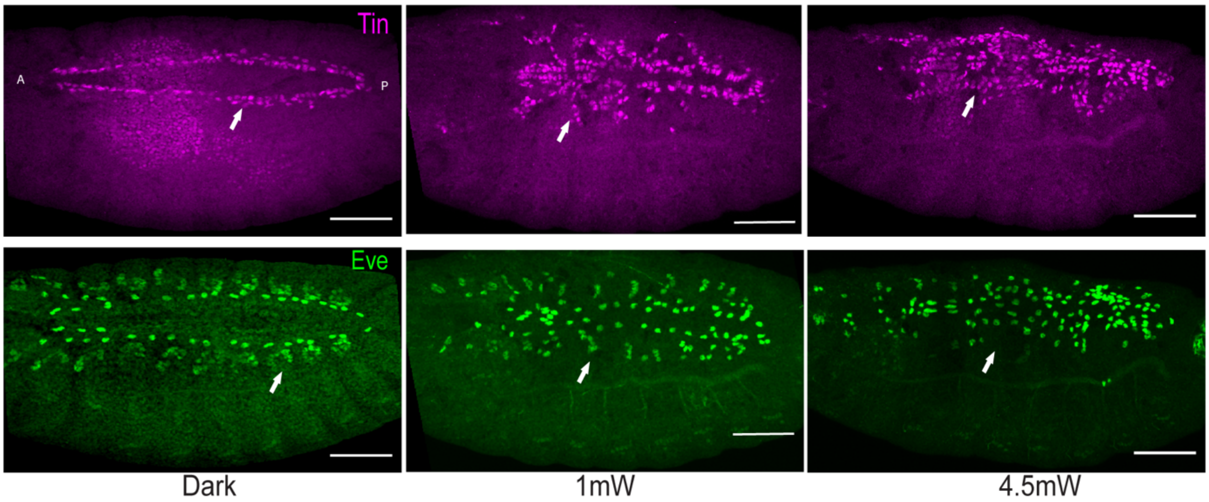

Supplementary Fig. 4 Opto-SOS activation in the mesoderm

Dorsal view of *twi*::Gal4>UAS-Opto-SOS embryos fixed and stained at stage 16 with Tin and Eve antibody. Embryos kept under dark show normal heart development with Tin and Eve patterns similar to wild-type. Embryos illuminated at 1mW and 4.5mW light intensity from stage 5 till stage 16 show a progressive increase in both Tin and Eve positive cells with a complete loss of the DA1 muscle group (arrowheads) at 4.5mW intensity. Scale bar = 50μm.

Supplementary Movie Legends:

Supplementary Movie 1: Opto-htl expression in the mesoderm during embryogenesis. *twi*::Gal4>Opto-htl embryo scanned with 561nm laser at 3 min intervals from stage 5 until end of embryogenesis to visualise expression of Opto-htl.

Supplementary Movie 2: Hatchability score for Opto-htl embryos under dark conditions. *twi*::Gal4>Opto-htl embryos imaged on a stereoscope covered with amber paper (to block 488nm light) from stage 5 until hatching. (Dark condition).

Supplementary Movie 3: Hatchability score for Opto-htl embryos under constant illumination. *twi*::Gal4>Opto-htl embryos imaged on a stereoscope from stage 5 until end of embryogenesis using a 488nm light intensity of 1mW measured at the sample plane. (Light condition).

Supplementary Movie 4: Cardioblast migration and matching in *hand*::GFP control embryos. *hand*::GFP control embryo imaged using a 488nm light-sheet to visualise the

migration of the GFP positive heart cells until matching at stage 16. (a) represents dorsal view; (b) represent lateral views of the same embryo.

Supplementary Movie 5: Hatchability score for WT (OreR) embryos under constant illumination. OreR embryos imaged on a stereoscope from stage 5 until end of embryogenesis using a 488nm light intensity of 1mW measured at the sample plane.

Supplementary Movie 6: Cardioblast migration and matching in Opto-htl embryo illuminated with 488nm laser. *twi*::Gal4>*hand*::GFP;Opto-htl embryos illuminated with 488nm laser from late stage 10 until early stage 13 and then imaged using a light-sheet microscope to visualise the migration of the GFP positive heart cells until matching at stage 16. (a) represents dorsal view; (b) and (c) represent lateral views of the same embryo.

Supplementary Movie 7: Cardioblast migration and matching in Opto-htl embryo kept under dark conditions. *twi*::Gal4>*hand*::GFP; Opto-htl embryo kept under dark condition until stage 13 and then imaged to visualise the migration of the GFP positive heart cells until matching at stage 16.

Sequences and Primers:

Myr signal sequence (src42A)

```
ATGGGTAACTGCCTCACCACACAGAAGGGCGAACCCGACAAGCCCGCA
```

Htl FGF cytoplasmic region (Amino acid position 331-729)
(Ref. http://www.uniprot.org/uniprot/Q07407)

```
TATGCCATCCGAAAGATGAAACATGAAAAGGTGTTGAAACAACGCATCGAAACCGTTCACCAGTGGACCAAGAAAGTGAT
CATCTTCAAGCCCGAAGGTGGCGGAGACTCCAGTGGTTCCATGGACACCATGATTATGCCGGTGGTTAGGATACAGAAAC
AGCGCACCACTGTTCTTCAGAATGGCAACGAGCCGGCTCCATTCAATGAATATGAATTTCCACTGGACTCGAACTGGGAA
CTGCCCAGAAGTCATTTGGTACTGGGTGCCACTTTGGGAGAAGGTGCTTTCGGACGAGTGGTCATGGCGGAGGTCAATAA
TGCCATTGTCGCCGTGAAAATGGTGAAGGAAGGACACACGGATGATGACATTGCCAGCTTGGTGCGGGAAATGGAAGTGA
TGAAGATCATTGGGCGACATATCAATATTATTAACTTACTTGGTTGCTGCAGTCAAAATGGTCCGCTCTATGTGATTGTC
GAGTATGCGCCACACGGAAATCTCAAGGACTTCCTCTATAAAAATCGACCCTTCGGAAGGGATCAGGATAGGGACAGCTC
GCAACCGCCGCCATCGCCACCAGCTCATGTGATAACCGAAAAGGATCTGATCAAATTTGCCCACCAAATTGCCAGAGGAA
TGGACTATTTGGCCTCGCGGCGATGCATCCATCGAGATTTGGCAGCCAGGAATGTGCTCGTCAGCGATGATTATGTGCTG
AAGATTGCTGATTTTGGACTGGCGAGGGACATTCAAAGCACGGATTACTATCGGAAGAACACAAATGGCAGGCTACCCAT
CAAATGGATGGCACCGGAGTCGCTGCAGGAGAAATTCTATGATTCCAAGAGCGATGTCTGGTCATATGGCATCCTGCTGT
GGGAGATCATGACCTATGGGCAGCAACCATATCCAACTATCATGTCCGCTGAGGAGCTGTACACCTATCTCATGTCCGGT
CAGCGGATGGAGAAACCAGCGAAATGCTCCATGAACATCTACATTCTGATGCGACAATGTTGGCATTTCAACGCCGACGA
TCGGCCACCTTTTACGGAAATTGTTGAGTATATGGACAAGCTGCTCCAGACGAAGGAGGACTACCTCGATGTGGATATCG
CCAATCTGGATACACCGCCCTCGACTAGCGACGAGGAGGAAGATGAAACGGACAACCTGCAGAAGTGGTGTAATTAT
```

CRY2mcherry (Ref. Addgene26866)

atgaagatggacaaaaagactatagtttggtttagaagagacctaaggattgaggataatcctgcattagcagcagctgc
tcacgaaggatctgtttttcctgtcttcatttggtgtcctgaagaagaaggacagttttatcctggaagagcttcaagat
ggtggatgaaacaatcacttgctcacttatctcaatccttgaaggctcttggatctgacctcactttaatcaaaacccac
aacacgatttcagcgatcttggattgtatccgcgttaccggtgctacaaaagtcgtctttaaccacctctatgatcctgt
ttcgttagttcgggaccataccgtaaaggagaagctggtggaacgtgggatctctgtgcaaagctacaatggagatctat
tgtatgaaccgtgggagatatactgcgaaaagggcaaaccttttacgagtttcaattcttactggaagaaatgcttagat
atgtcgattgaatccgttatgcttcctccttggcggttgatgccaataactgcagcggctgaagcgatttgggcgtg
ttcgattgaagaactagggctggagaatgaggccgagaaaccgagcaatgcgttgttaactagagcttggtctccaggat
ggagcaatgctgataagttactaaatgagttcatcgagaagcagttgatagattatgcaaagaacagcaagaaagttgtt
gggaattctacttcactactttctccgtatctccatttcggggaaataagcgtcagacacgttttccagtgtgcccggat
gaaacaaattatatgggcaagagataagaacagtgaaggagaagaaagtgcagatctttttcttaggggaatcggtttaa
gagagtattctcggtatatatgtttcaacttcccgtttactcacgagcaatcgttgttgagtcatcttcggtttttccct
tgggatgctgatgttgataagttcaaggcctggagacaaggcaggaccggttatccgttggtggatgccggaatgagaga
gctttgggctaccggatggatgcataacagaataagagtgattgtttcaagctttgctgtgaagtttcttctccttccat
ggaaatggggaatgaagtatttctgggatacacttttggatgctgatttggaatgtgacatccttggctggcagtatatc
tctgggagtatccccgatggccacgagcttgatcgcttggacaatcccgcgttacaaggcgccaaatatgacccagaagg
tgagtacataaggcaatggcttcccgagcttgcgagattgccaactgaatggatccatcatccatgggacgctcctttaa
ccgtactcaaagcttctggtgtggaactcggaacaaactatgcgaaacccattgtagacatcgacacagctcgtgagcta
ctagctaaagctatttcaagaacccgtgaagcacagatcatgatcggagcagcaGCCCGGGATCCACCGGTCGCCACCAT
GGTGAGCAAGGGCGAGGAGGATAACATGGCCATCATCAAGGAGTTCATGCGCTTCAAGGTGCACATGGAGGGCTCCGTGA
ACGGCCACGAGTTCGAGATCGAGGGCGAGGGCGAGGGCCGCCCCTACGAGGGCACCCAGACCGCCAAGCTGAAGGTGACC
AAGGGTGGCCCCCTGCCCTTCGCCTGGGACATCCTGTCCCCTCAGTTCATGTACGGCTCCAAGGCCTACGTGAAGCACCC
CGCCGACATCCCCGACTACTTGAAGCTGTCCTTCCCCGAGGGCTTCAAGTGGGAGCGCGTGATGAACTTCGAGGACGGCG
GCGTGGTGACCGTGACCCAGGACTCCTCCCTGCAGGACGGCGAGTTCATCTACAAGGTGAAGCTGCGCGGCACCAACTTC
CCCTCCGACGGCCCCGTAATGCAGAAGAAGACCATGGGCTGGGAGGCCTCCTCCGAGCGGATGTACCCCGAGGACGGCGC
CCTGAAGGGCGAGATCAAGCAGAGGCTGAAGCTGAAGGACGGCGGCCACTACGACGCTGAGGTCAAGACCACCTACAAGG
CCAAGAAGCCCGTGCAGCTGCCCGGCGCCTACAACGTCAACATCAAGTTGGACATCACCTCCCACAACGAGGACTACACC
ATCGTGGAACAGTACGAACGCGCCGAGGGCCGCCACTCCACCGGCGGCATGGACGAGCTGTACAAGTAA

Primers for amplifying FGF cytoplasmic region

FP 5′- GAACCCGACAAGCCCGCATATGCCATCCGAAAGATGAAAC -3′

RP 5′-ctttttgtccatcttcatATAATTACACCACTTCTGCAGGTTGTC-3′

Primers for amplifying CRY2-mcherry

FP- 5′-CAGAAGTGGTGTAATTATatgaagatggacaaaaagactatagtttgg-3′

RP 5′- GGGGTGCCTAATGCGGCCGCTTACTTGTACAGCTCGTCCATGC -3′

Myr sequence synthesized

5′-GGTATACACCTAGGCGGTACCATGGGTAACTGCCTCACCACACAGAAGGGCGAACCCGACAAGCCCGCATATGCCATCCG
AAAGATG-3′

3′-CCATATGTGGATCCGCCATGGTACCCATTGACGGAGTGGTGTGTCTTCCCGCTTGGGCTGTTCGGGCGTATACGGTAGGC
TTTCTAC-5′
